# Nurse Task Shifting for Antiretroviral Treatment Services in Namibia: Implementation Research to Move Evidence into Action

**DOI:** 10.1371/journal.pone.0092014

**Published:** 2014-03-18

**Authors:** Gabrielle O’Malley, Lily Asrat, Anjali Sharma, Ndapewa Hamunime, Yvonne Stephanus, Laura Brandt, Deqa Ali, Francina Kaindjee-Tjituka, Salomo Natanael, Justice Gweshe, Caryl Feldacker, Ella Shihepo

**Affiliations:** 1 International Training and Education Center for Health, University of Washington, Seattle, Washington, United States of America; 2 International Training and Education Center for Health, University of Washington, Windhoek, Namibia; 3 Namibia Ministry of Health and Social Services, Windhoek, Namibia; UCL Institute of Child Health, University College London, United Kingdom

## Abstract

**Background:**

Evidence from several sub-Saharan countries support nurse-initiated antiretroviral treatment as a feasible alternative to doctor-led models characteristic of early responses to the HIV epidemic. However, service delivery models shown to be effective in one country may not be readily adopted in another. This study used an implementation research approach to assist policy makers and other stakeholders to assess the acceptability and feasibility of task shifting in the Namibian context.

**Methods:**

The Namibian Ministry of Health and Social Services implemented a Task Shifting Demonstration Project (TSDP) at 9 sites at different levels of the health system. Six months after implementation, a mixed methods evaluation was conducted. Seventy semi-structured interviews were conducted with patients, managers, doctors and nurses directly involved with the TSDP. Physician-evaluators observed and compared health service provision between doctors and nurses for 40 patients (80 observations), documenting performance in agreement with the national guidelines on 13 clinical care indicators.

**Results:**

Doctors, nurses, and patients interviewed believed task shifting would improve access to and quality of HIV services. Doctors and nurses both reported an increase in nurses’ skills as a result of the project. Observation data showed doctors and nurses were in considerable agreement (>80%) with each other on all dimensions of HIV care and ≥90% on eight dimensions. To ensure success of national scale-up of the task shifting model, challenges involving infrastructure, on-going mentoring, and nursing scope of practice should be anticipated and addressed.

**Conclusion:**

In combination with findings from other studies in the region, data from the TSDP provided critical and timely information to the Namibian Ministry of Health and Social Services, thus helping to move evidence into action. Small-scale implementation research projects enable stakeholders to learn by doing, and provide an opportunity to test and modify the intervention before expansion.

## Introduction

Namibia faces a critical shortage of medical doctors, approximately 3 doctors per 10,000 population, and is heavily reliant upon foreign health care workers to support public sector services. [1] As national HIV clinical guidelines stipulate that only medical doctors can initiate patients on antiretroviral treatment (ART), these few highly skilled healthcare workers have been tasked with providing quality care for patients in the context of a generalized HIV epidemic, hovering around 18% prevalence. [2] Access to this life-saving ART remains unequally distributed. The Namibian Health Facility census of 2009 showed that only 18% of facilities in the country offered ART services, with the majority of services available in hospitals [3].

Within this challenging environment, Namibia successfully scaled up key HIV clinical services, with estimated national ART and prevention of maternal-to-child transmission coverage over 80%. [3] However, the existing physician-centered ART care model cannot adequately deliver quality clinical services to the over 100,000 Namibians currently on ART, nor the tens of thousands of additional HIV-infected persons who may become eligible for ART by 2014/15. [2], [4], [5] Currently, many sub-district health facilities do not have a physician on site and are instead reliant upon periodic outreach visits for initiation and monitoring of ART. In health facilities that have dedicated HIV physicians, ART clinics are often crowded and have long patient wait times. [5] ART patients may have to travel long and costly distances to a doctor-staffed clinic or make appointments only on a specific physician-visit day. In some more remote locations, anecdotal reports suggest that nurses who wish to meet HIV-infected patients’ needs sometimes initiate and monitor ART for patients even though they are untrained in ART initiation. These factors further restrict access to quality health care services for Namibia’s population.

Recent policy changes have further driven demand which could potentially overload a healthcare system already at capacity. [4] In 2010, national HIV treatment eligibility criteria changed so that patients could begin ART earlier in the progression of the disease (at a CD4 count of 350 cells/mm^3^ instead of 200 cells/mm^3^). The Ministry of Health and Social Services (MOHSS) has also committed to future implementation of “treatment as prevention,” including universal ART for all HIV-infected pregnant women and expanded eligibility for serodiscordant couples. [4], [6] Although favorable policies should increase demand for, and access to, ART, limited geographic availability of ART and severe shortages of medical personnel to administer it may threaten successful implementation and the quality of patient care.

The Ministry of Health and Social Services in Namibia recognized the critical need for timely solutions to meet the anticipated increase in ART demand with high quality, accessible services across a large geographic area. In 2009 when this evaluation was planned, there were already positive reports from other countries on the use of task shifting for HIV care and treatment. [7]–[19]. While recognizing the positive implications these findings could have for the challenges facing HIV service delivery in Namibia, the MoHSS was not convinced that data emerging from other countries necessarily meant similar task shifting efforts would result in positive outcomes in their own context. Rather, the MoHSS wanted to determine whether task shifting of ART patient initiation and care from medical doctors to nurses would work in their own setting using their own healthcare workers. In addition, they believed that data generated from their own country would be more powerful in advocacy efforts to change policy and practice expectations if warranted. Therefore, the MoHSS asked International Training and Education Center for Health (I-TECH) to help them implement a small demonstration project to assess comparability of care between doctors and nurses, acceptability of nurse initiated ART, and feasibility of task shifting within the Namibian national health care context.

The Task Shifting Demonstration Project (TSDP) was conceived as a small implementation research project aimed to promote uptake and successful implementation of an evidence based service delivery model, [20] and was designed to address several key considerations and constraints. First, as nurses were not previously trained nor allowed by the Namibian Nursing Scope of Practice to initiate ART treatment, a large scale study of the intervention would have required changes in the national regulatory framework and the health systems supporting HIV services. The MoHSS did not want to advocate for this step without any data from Namibia. Therefore, the pilot project trained only a small number of nurses from different levels of the healthcare system to test the approach under strict monitoring. Second, to ensure quality patient care, nurses would only be trained and expected to initiate and monitor ART for stable patients without significant HIV-associated co-morbidity, and to recognize and refer patients with more complicated HIV disease. Third, cost constraints were taken into account by limiting the geographic distribution of selected sites. Lastly, a rapid assessment and report on training, implementation, and outcomes was requested in order to respond to urgent needs of expanded ART service delivery. If successful, the MoHSS planned to immediately use the results to advocate for changes in nursing scope of practice and to expand training for task shifting, rather than to wait for formal publication of the results. Given reports from elsewhere in sub-Saharan Africa, the expected acceptability of the practice and quality of nurse-provided care in Namibia would also make further delays in implementation of the intervention unethical.

The TSDP was commissioned as an urgently needed intervention, implemented with a small group of nurses in multiple, geographically dispersed sites with high levels of institutional support. This implementation research approach, rather than a large, generalizable research study, is a well-documented strategy in the literature that allows for more responsiveness to programmatic, resource, and time constraints and provides stakeholders with the information necessary to confidently put “evidence into action. [21]–[28] It was expected that the results of this small pilot, if positive, would add credibility to the task shifting approach in general, demonstrate potential replicability of findings within the Namibian context, and inform training and other programmatic implementation challenges likely to be encountered if scale-up were to occur.

## Methods

### Aim and Objectives

The overarching goal of the TSDP was to assess the feasibility of a task shifting approach from nurses to doctors in patient initiation of ART in Namibia. The MoHSS was specifically interested in knowing whether shifting ART initiation and provision from doctors to nurses for patients with uncomplicated HIV disease would be acceptable to patients, nurses and doctors; whether stable HIV positive patients would receive a comparable level of care from nurses as they would from doctors; and if a task shifting approach were to be implemented, what challenges and opportunities might they encounter at different levels of the health care system. All steps of the process, including intervention design, training participant selection, site selection and evaluation methods, were implemented in close consultation with the MoHSS.

### Site Selection

**Figure 1:**
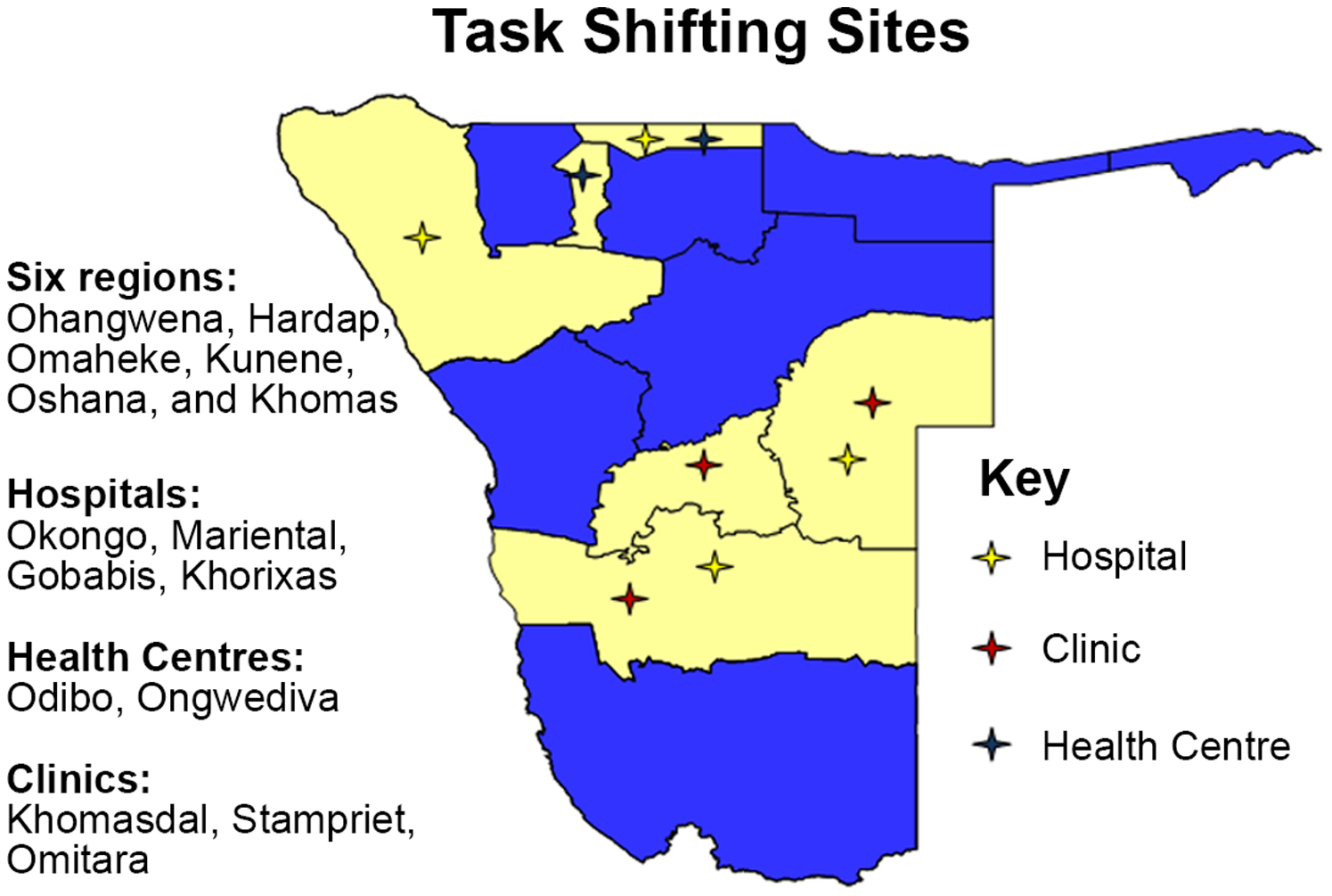
Task shifting demonstration project sites in Namibia.

Nine sites were purposively selected to provide a wide spectrum of information on the opportunities and constraints on which to base recommendations for implementing task shifting across Namibia. Selected sites met the following requirements: a) included different levels of the health care system (hospital, health center, clinic); b) were supported by the existence of a district hospital and were within reasonable distance for patient referrals; c) had access to a laboratory for patient follow-up in biochemistry and hematology (either on-site or within reasonable distance to a regional laboratory); d) had adequate stocks of essential supplies such as cotrimoxazole; e) included at least six of the thirteen regions in Namibia; (f) had access to an I-TECH clinical mentor, an identified nurse mentor, or district supervisor to provide supervision and support g) were geographically accessible to evaluation staff to ensure careful monitoring and evaluation of the Task Shifting Model. Therefore, the pilot project trained only a small number of nurses from different levels of the healthcare system, in six different regions of the country, to test the approach under strict monitoring (Figure 1). The number of patients on ART at each of the selected sites at the time of the demonstration project is indicated in Table 1. Before the task shifting project, ART was generally only initiated at the clinics during “outreach day” when a visiting physician was available. Therefore, the number of ART patients at the clinic sites is small.

**Table 1:** Task Shifting Sites: Number of Patients on ART as of December 31, 2010*.

Health Facility	Region	# Patients on ART
Stampriet Clinic	Hardap	45
Mariental Hospital	Hardap	436
Khorixas Hospital	Kunene	318
Omitara Clinic	Omaheke	45
Gobabis Hospital	Omaheke	965
Odibo Health Center	Ohangwena	962
Ongwediva Health Center	Oshana	1,111
Okongo Hospital	Ohangwena	1,196
Khomasdal Clinic	Khomas	430
Total		5508

*Data source: Namibia National Data Base (NDB).

### Site Preparation: Training and Mentorship

In the regions where demonstration sites were selected, Regional Directors were requested to release one or two nurses per site for the task shifting training who were currently providing ongoing care to patients with HIV, and who would not be moved from the ART clinic for the six-month duration of the demonstration project. In July 2011, a total of eleven nurses from nine facilities received one week of classroom training and one week of applied clinical training in a high-volume facility. Simultaneously, seven doctors underwent a three-day training in clinical mentorship. Following the intensive two-week training, nurses were to receive ongoing mentorship support from one of these trained ART physician-mentors for the duration of the demonstration project. Some mentors worked at the same site as the nurses, i.e. at the hospital level, while others did not. i.e. at the clinic level. Off-site ART doctors provided mentorship over the phone and during outreach visits.

### Implementation Monitoring

The six-month demonstration project was purposively designed to minimize interruption to routine patient services and human resource staffing. Patient monitoring and initiation on ART at the pilot sites continued to be undertaken by on-site or visiting doctors, as well as trained nurses. In these routine practice settings, patients were sometimes seen by doctors, sometimes by nurses, and sometimes by both. Study staff contacted participating nurses and doctors by phone at two- and four-weeks post-training, and conducted site visits at six weeks post-training to assess whether nurses had begun ART initiation and management of stable patients, whether they were receiving mentoring support from the physician mentors, and to identify any other challenges nurses were facing. Where possible, evaluation staff worked with the Deputy of Special Programs at the MoHSS to address identified challenges.

### Evaluation

In February 2011, approximately six months after the task shifting training, the acceptability, comparability of care, and feasibility of the task shifting model was assessed by two evaluation teams using mixed-methods. Each team consisted of a team lead highly experienced in qualitative methods, a physician-evaluator with expertise in HIV clinical management, and an interviewer fluent in English and at least one local language.

The teams conducted a total of 70 semi-structured face-to-face qualitative interviews with patients, regional and district managers, nurses, and doctors to assess perceived effectiveness and acceptability of task shifting [Table 2]. Interview teams were led by highly experienced qualitative researchers (LA, AS). An open-ended, pilot-tested interview guide was used to ensure consistency across interviews. All participants provided written informed consent in the language of their choice. Interviews were conducted in private spaces at the health facilities and digitally recorded. Interviewers documented their observations and reflections of the interview session. Patient interviews were conducted in English or one of the local languages by study assistants who were trained and supervised by LA and AS. Interviews were transcribed and translated if the interview was conducted in a language other than English. A convenience sample of patients waiting to be seen in the HIV clinic on the days the study team was present were recruited. Eligibility criteria included being over 18, feeling well enough to be interviewed, and having attended the clinic for at least seven months. In some instances, initial contact with patients was by members of the study team, and in some cases initial contact was by the HIV clinic nurse-receptionist. Nurse-receptionists did not keep track of the number of patients approached who refused to learn more about the interview process. There were no reported refusals among patients approached by study team members. No patients refused to be interviewed during informed consent. Patient interviews lasted between approximately 10–15 minutes.

**Table 2:** Number of Interviews by Site.

Site (N = 9)	Regional/District Manager (N = 8)	Site Manager (N = 7)	Doctor (N = 7)	Nurse (N = 11)	Patient (N = 39)
Gobabis	1	1	1**	1	6
Khomas	1	1	1	1	6
Khorixas	1	1	1**	2	6
Mariental	3	1	1**	1	6
Odibo	–	1	1	1	5
Okongo	1	1	1	1	4
Omitara* ^+^	–	–	–	1	–
Ongwediva	1	1	1	2	6
Stampriet* ^◊^	–	–	–	1	–

*No patient interviews were conducted at these sites since task shifting either hadn’t been implemented, or hadn’t been implemented long enough for patients to have had multiple visits for HIV care at that facility.

**This individual is also the Site Manager.

*^+^All interviews covered under Gobabis Hospital/Omaheke Region.

*^◊^All interviews covered under Mariental Hospital/Khorixas Region.

Evaluation team leaders (LA, AS) interviewed regional, district, and clinic managers involved with the TSDP to assess their views on the advantages and disadvantages of the nurse-initiated ART model and the feasibility of scaling up the task shifting project. Doctors and nurses were interviewed to gather their perceptions of the impact of the TSDP on the quality of care at their sites and advisability of scaling-up the model to other sites across Namibia. No managers or site-level health care workers refused to be interviewed and all were interviewed in English by LA and AS. Manager and Health care worker interviews varied widely, between approximately 20–60 minutes.

Physician-evaluators conducted 40 paired doctor and nurse observations (N = 80 observations) of six of seven doctors and ten of eleven nurses [Table 3] trained as part of the TDSP in order to compare quality of care provided by nurses and doctors, and to assess whether nurses were able to discern when to refer patients to a higher level of care. One nurse and one doctor were not eligible for inclusion in the paired observation analysis because the ART doctor was unavailable. Structured observation instruments designed to follow the flow of clinical encounters were used to document the patient-provider consultation. The observation instrument was adapted to the Namibian context from an evaluation of HIV clinical care provided by mid-level health care workers in Mozambique [29].

**Table 3:** Location of Task Shifting Doctors and Nurses.

Region (N = 6)	Health Facility (N = 9)	Location of Assigned doctors/mentors (N = 7)	Location of Nurses (N = 11)
Hardap	Stampriet Clinic	Mariental Hospital	1
	Mariental Hospital		1
Kunene	Khorixas Hospital	Khorixas Hospital	2
Omaheke	Omitara Clinic	Gobabis Hospital	1
	Gobabis Hospital		1
Ohangwena	Odibo Health Centre	Odibo Health Centre	1
	Okongo Hospital*	Okongo Hospital*	1
Oshana	Ongwediva Health Centre	Oshakati Intermediary Hospital	2
Khomas	Khomasdal Clinic	Katutura Health Centre	1

*No paired observation possible between doctor and nurse at this hospital.

A stratified purposive sample of patients seeking care on the day the evaluation team was present were asked to verbally consent to the observation in English, Afrikaans, and Oshiwambo as per language preference. At each facility, recruitment efforts targeted two pre-ART patients (CD4>350 cells/mm3), two patients ready to initiate ART, and two follow-up patients. Patients who agreed to the clinical consultation observations were randomly assigned to first see the doctor and then the nurse, or vice versa. The doctor and nurse were asked to follow normal practice but withhold the diagnosis and treatment plan from the patient until after both consultations. Physicians and nurses were blinded to each other’s consultations. The physician-evaluators observed the consultations, sought clarification in private brief interviews with either the doctor or the nurse as necessary, and completed the observation form, noting both what the health care worker did and whether or not it was consistent with national clinical guidelines for HIV care and treatment, given specific site considerations (i.e. whether or not diagnostic materials and supplies were in place). For example, “correct” management of an HIV-infected patient with ascites may consist of referral to a higher level of care if patient attended a rural facility, or immediate paracentesis and abdominal ultrasound if patient attended a referral hospital.

### Data Quality Assurance

The evaluation team leaders conducted a two-day training for the physician evaluators and interviewers on recruiting, consenting, maintaining confidentiality, and data collection and safety. The training included a detailed review of all materials such as observation method, careful/accurate observations, note taking, facilitation, and techniques for ensuring data quality. Practice and problem solving on interviews and observations were used to standardize approaches and to increase inter-physician evaluator reliability. Team leaders provided on-going monitoring of data collection activities in the field and reviewed all completed instruments daily for legibility and completeness. Ambiguous information from interview notes and on observation forms was clarified. Ten percent of the quantitative data was double entered and discrepancies resolved via comparison to the observation forms.

### Data Analysis

For the qualitative analysis, preliminary codes were generated from the evaluation questions and assigned to relevant text in the interview transcripts by GO and a study assistant. Coded text and suggested new codes were compared and discussed between the initial coders until agreement was reached. The coding structure was then applied to all interview transcripts in ATLAS.ti, which were further analyzed by GO for sub-themes through an iterative process. [30], [31] Interview data were analyzed by site and then across sites, and between health care workers and patients, to compare perceptions on the acceptability of the TSDP, as well as to identify common and site-specific challenges. The interpretation and synthesis of qualitative findings was circulated among evaluation team members (LA, AS, YS, LB, NH) for discussion until consensus was reached.

Quantitative data from clinical observations reflecting 13 quality of care indicators were analyzed using descriptive analysis of service provision in SPSS 18.0 and STATA 11 and binomial confidence intervals were calculated [Table 4]. Our analysis considered the judgment of the physician-evaluators to represent the ‘gold standard’ to which both the task-shifting doctor and nurse were compared. The physician-evaluators based their assessments on the Namibian national guidelines. Our analysis included three different comparisons: the doctor to the physician-evaluator, the nurse to the physician-evaluator, and the doctor and nurse to each other. Comparing the task shifting doctor and nurse to each other only would not have told us whether the practice of either was meeting the national guidelines.

**Table 4:** Agreement Found During Clinical Observations (95% CI).

			Column A:	Column B:	Column C:
Dimensions of clinical care in accordancewith the national guidelines:	N = total observations	N = Paired observations	% Doctor followednational guidelines	% Nurse followednational guidelines	% AgreementDoctor and Nurse
1. Conducted thorough history relevant to current symptom/complaint	80	40	97.5% (CI.87, 1)	97.5% (CI.87, 1)	100% (CI.91,1)
2. Accurately identified ART eligibility	48	24	87.5% (CI.68,.97)	87.5% (CI.68,.97)	83.3% (CI.63,.95)
3. Recommended appropriate laboratory tests	80	40	92.3% (CI.8,.98)	92.5% (CI.8,.98)	95% (CI.83,.99)
4. Accurately diagnosed opportunistic infections	80	40	100% (CI.91, 1)	97.5% (CI.87,1)	97.5% (CI.87, 1)
5. Accurately interpreted laboratory values	80	40	100% (CI.91,1)	97.5% (CI.87, 1)	97.5% (CI.87, 1)
6. Accurately assessed WHO clinical stage	80	40	97.5% (CI.87, 1)	92.5% (CI.80,.98)	95% (CI.83,.99)
7. Recommended appropriate ART medication	80	40	95% (CI.83,.99)	92.5% (CI.80,.98)	92.5% (CI.80,.98)
8. Appropriately documented consultation findings	80	40	90% (CI.76,.97)	95% (CI.83,.99)	92.5% (CI.8,.98)
9. Adequately addressed ART adherence	32	16	100% (CI 79, 1)	87.5% (CI.62,.98)	87.5% (CI.62,.98)
10. Accurately diagnosed side effects	32	16	100% (CI.79,.1)	87.5% (CI.62,.98)	87.5% (CI.62,.98)
11. Accurately diagnosed other conditions	80	40	87.5% (CI.73,.96)	77.5% (CI.61,.89)	85% (CI.70,.94)
12. Physical examination in relation to history and current complaint	80	40	77.5% (CI.62,.89)	67.5% (CI.51,.81)	90% (CI.76,.97)
13. Accurately assessed for treatment failure	32	16	68.8% (CI.41,.89)	62.5% (CI.35,.85)	81.3% (CI.54,.96)

### Ethical Review

The Task Shifting Demonstration Project and publication of the results was approved by the Office of the Permanent Secretary of the Ministry of Health and Social Services in Namibia. This activity was determined to be non-research by the University of Washington human subjects review board as its findings were not intended to be generalizable.

## Results

### Acceptability of Task Shifting – Patient Perspectives

Nearly all of the patients interviewed were in favor of task shifting with only one out of thirty-nine patients expressing reservations. Patients generally did not anticipate any negative changes in the quality of care they received or would receive in a nurse-led ART delivery model, and in fact, perceived several advantages. The following are typical responses of patients to interviewer questions about their experience at the demonstration sites.

When asked how care provided by a nurse differed from care provided by a doctor, patients often reported that there would be no difference:

*“From the time I started I can say it is now about two years that I haven’t been seen by a doctor. It was just the nurse that helps me …. like the one who helped me today, is the one that always helps me.”* [Gobabis patient 6]

*“I won’t see a difference between a nurse and a doctor because the medication that I get from a doctor is the same as the one I get from a nurse.”* [Ongwediva patient 3]

Patients also expressed confidence that trained nurses would know when they needed to refer cases to the doctor:

*“Sometimes when I* [am] *very sick the nurse can tell me that I can only be treated by the doctor because she also doesn’t know how to deal with that certain condition.”* [Odibo patient 6]

Patients perceived three major advantages to nurses playing a larger role in providing HIV care and treatment. One advantage is a reduction in wait time:

*“I would feel good about it* [trained nurse taking care of needs rather than a doctor] *because sometime the doctor is busy and a person has been waiting a long time and still* [has] *to wait long to get help. But the* [task shifting] *means one wouldn’t take long or sit in line again, waiting for help.”* [Gobabis patient 5]

A second advantage was that patients thought that task shifting was important for reducing time spent travelling to the health care facility:

*“Since doctors are fewer than nurses, it* [task shifting] *is fine so that we do not have to come back to the clinic to see the doctor the day when he is there.”* [Okongo patient 4]

Finally, patients reported that task shifting might improve communication between patients and HCWs since nurses were more likely than doctors to speak the patient’s language:

*“Nurses are nice because they can advise patients but doctors do not really advise because they do not speak Oshiwambo. And if he wants to do it* [advise], *he and the patient may not understand each other.”* [Odibo patient 3]

The one patient who responded negatively to having trained nurses rather doctors care for patients reported concern that doctors would need to be available, and emphasized nurse’s role in referral:

*“There are times when a person needs a doctor, there is no way that one would not need a doctor …. The doctor has to be higher than the nurse. They are the same in a way but sometimes the medication that I’m taking could bring me problems, in that case I have to speak to a doctor.”* [Mariental patient 5]

Even this patient qualified:

*“It all depends. If you need to see a doctor, they will send you to see a doctor. And if you don’t need to see a doctor, the nurse will see you.”*

### Health Care Worker Support of Task Shifting

Similarly, analysis of the interviews with health care workers and managers showed strong support for national scale-up of the nurse-led service delivery model for a variety of reasons. TSDP participants consistently reported an improvement in nursing skills.

*“I am now able to examine and even initiate patients on ART… I am doing physical exam, am able to assess patients and also diagnose…. I know how to start patients on ART now. If I saw results than I would know that I can start this patient on this treatment. And sometimes it was like you saw this patient is positive and you just knew we have to start them on ART but you would not know what regimen to choose. Actually it was only the doctors who knew. But now with task shifting we know.”* [Nurse 6]

*“She* [the nurse] *has improved a lot, improved a lot. First of all, you know the history taking? …before she can take the history but she will not* [be] *precise. Let’s say if someone is coughing, ‘For how long? When did you go for sputum’ … But most importantly, the thing that she really is doing now like almost for all patients is the physical examination, that most of the* [other] *nurses don’t do. They take the history–‘Okay, go to see the doctor.’ But now, she is putting the patient on the couch and examines them from head to toe, which is completely a new ability … A new skill for her … Even if I am not with her, if she hears something abnormal, she will come quickly … ‘I hear something like this, can you come and confirm’ … Something like that.”* [Doctor 4]

Even though some of the nurses had been providing the same *kind* of services to HIV patients before the TSDP, nurses, doctors, and managers reported that after the task shifting training, they were providing those services better:

*“The difference is … They are improved now … They* [had] *started doing the things, without the proper training. Now they have been trained, they have improved. Yeah … they have been doing it* [initiating and managing patients with HIV] *but now they are doing it much, much better. And, they have become more efficient in any case.”* [Doctor 2]

With nurses taking on a broader role in initiating HIV patients on ART, doctors reported an increased ability to focus on complicated patients. For example:

***“*** *I cover many departments in the hospital. In the past it has been quite a stressful situation because I have had to run up and down to different departments to try to cover all my duties. And sometimes the quality of service delivery becomes restricted because you are in the rush all the time. With the task shifting I have appreciated that at least the work pressure is reduced a bit, especially with regards to ART clients. Because the clients that would wait for me for a long period of time, some of them have already been attended to by the time I come to the ART clinic. So that has been a very positive aspect. And for the few clients that remain, I have more time to do a detailed assessment on them. And then it also gives me more time to do other duties in the hospital. So I think it has been positive overall.”* [Doctor 6]

Several HCWs mentioned that the redistribution of tasks from doctors to nurse has resulted in an improvement in patient flow which has been a benefit to the whole clinic. For instance:

*“Other staffs are also happy with this project. I can give an example of the pharmacy …before this task shifting project, it would mean that the doctor is the one seeing all the patients. I am also supposed to be doing ward rounds in the general hospital apart from this clinic. You would find that as long as I was tied up with one emergency or the other at the hospital, those patients would be kept waiting. But this project means that even when the doctor is not there, patients are now being seen. There is no disruption in the flow of patients. It also means that the pharmacist is also happy as he is seeing patients as they are coming rather than to wait for the doctor to come and then see patients at one go. So it has also improved patients waiting times, so I think every department is happy with that.”* [Manager/doctor 9]

Perceptions were widespread among nurses, doctors and health facility managers that with nurses being able to handle more of the patient load, patient wait time at the clinic had decreased. For example:

*“Initially it used to be the doctor* [reviewing patients] *so we would just assess. We would collect the blood, do the BP* [blood pressure], *the parameters, then, we’d just take the files. They* [files] *wait for the doctor. So by the time the doctor comes from the medical wards, that’s when he is going to see those patients. And you would find cases where there are many or maybe 20 or 30 waiting for him. But with the coming of this task shifting, patients are no longer waiting. We only refer the complicated cases. So I have seen a very big development in that patients no longer wait for long periods as they used to do.”* [Nurse 10]

HCWs also believed that if they did not have to wait to be seen by a doctor, patients would be more likely to initiate ART in a timely manner:

*“For patients, they are getting proper care. They are not delayed on ARV because they have to be pushed to outreach day. Now they can come any day. I can take care of patients.”* [Nurse 1]

*“I think the, with this project in place, more and more clients will be started as early as possible because we don’t have enough doctors in our country. Otherwise, if we rely on the doctors, then most of the AIDS patients will suffer. They will be initiated very late and then, some will even die. But for this project now, this task shifting, it is really a necessity for a patient…. They will survive.”* [Manager]

Although happy about their increased capacity to of provide a higher level of care to their patients, some nurses also reported concern about their increased workload.

*“To me it is a challenge because there is a lot on my side compared to the manpower that is available because I am also seeing ordinary patients and sometimes there’s too much work on me and the HIV patients I might not give them the full attention which is needed. So if they would add one more nurse, maybe will be better.* [Nurse 9]

Some sites addressed this workload by re-distributing some of the nurse’s tasks to others. One nurse described the redistribution as follows:

*“Okay, since some of the tasks are shifted to me, I also started shifting some tasks to my subordinates. And so it has also increased supervision to them because I have to be concerned that they are really doing things as they are supposed to be done, yeah …….* [now they attend to] *the PMTCT mothers. I used to do the counseling and so on – so, I have shifted down to some of the nurses. Like the completion of the registers, the ART registers for the test for the babies. So the paperwork that was there, I have shifted to the clerks and the counsellors … They are doing fine … They don’t complain because they also do appreciate what I am doing because they see that it is really helping the patients. So they are taking it in that line it’s really helpful.”* [Nurse 5]

An important component of this demonstration project was training doctors on good mentoring practices. Doctors expressed satisfaction with what they had learned in the mentoring training and liked the improved mentoring relationship with the nurses.

*“I think I really benefitted a lot from it* [the training] *because even in my experience when I was working with the task shifting nurses, there are times when we want to communicate. This training really equipped me with basic communication skills of how to get information, how to give feedback, and how to correct and how to guide. At the same time, there are also aspects that the mentoring process also was a learning process for me. It also helped me to self-evaluate myself through mentoring my nurses.”* [Doctor 6]

Nurses also appreciated the mentoring relationship with the doctors.

*“Sometimes.. if our doctor is there, if he sees something interesting then he will invite me. Sometimes if I come across something interesting, I call* [the doctor] *and then he will ask me some questions, ask me what I think, add a few more things …. It stimulated me to really try and think what management I can give. This was a change from before …. like normally the doctor was just looking and telling you to do the management. But for him, now he is trying to find out from me what I know, what I think, … it is really stimulating me to think about what I should do for my patients.”* [Nurse 1]

Both doctors and nurses thought on-going mentoring support would be important so that nursing clinical skills could continue to improve and so they could manage more complicated patients. However, time constraints were perceived to be a potential challenge to scaling up the task-shifting model.

*“The thing that still remains is that we don’t give it* [mentoring] *enough time because half a day, you are just too tempted just to finish seeing all the patients. The temptation is there …. and I have patients here too at the main hospital. … Even sometimes when you see a challenging case …. you are presented with a good teaching opportunity, which just because of time, you say ‘Ah no, let me leave it because time is not on my side’.”* [Doctor 2]

In addition to anticipated challenges of consistent mentoring support for nurses in an expanded model, interview and implementation monitoring data collected by the evaluation team also identified other challenges to a nationwide scale-up of the TSDP. Although nurse initiated ART was successfully implemented at eight of the nine demonstration sites, one of the clinics was not able to change over to a nurse-led model from an outreach model (monthly visits by doctor) within the six-month long task shifting demonstration. Moving away from the outreach model which had been used at the clinics required systems changes (e.g. patient records being kept on-site rather than with the outreach team, the outreach doctor continuing to initiate most of the patients himself) which were not completed by the time of the final evaluation. Another observed and reported challenge was lack of clinic space when doctors rotating into the site occupied the only available exam room.

### Comparability of Care – Quantitative Data

Results from structured observations of clinical practice of nurses and doctors involved in the TSDP are shown in Table 4. The number of observations varied across dimensions of clinical care because not all dimensions of clinical care were relevant to all patients. For example, treatment failure could not be assessed in cases where a patient was ART naïve. Results showed considerable agreement (>80%) with each other on all dimensions of HIV care and ≥90% on eight dimensions (Table 4, Column C). These percentages reflect agreement whether or not the observed care was in line with the national clinical guidelines. A more nuanced description of clinical care can be seen by the proportion of cases wherein doctors and nurses performed in accordance with national clinical guidelines, operationalized as being in agreement with the physician-evaluator (Table 4, Columns A and B). On three dimensions of clinical care (conducting thorough history, accurately identifying ART eligibility, and recommending appropriate laboratory tests) doctors and nurses followed national guidelines on the same number of cases. Doctors followed national guidelines on 3% more cases than nurses on three dimensions of clinical care (diagnosing opportunistic infections, recommending appropriate ART medication, and accurately interpreting laboratory values) and on 5%–10% more cases than nurses on three other dimensions of clinical care (adequate physical exam, accurately assessing the WHO stage, accurately diagnosing non-HIV related conditions). Nurses scored higher than doctors on appropriately documenting consultation findings in 5% of cases. The largest difference in performance between doctors and nurses was in accurately diagnosing side effects, and adequately addressing ART adherence. For both of these dimensions, doctors followed national guidelines in 100% of cases and nurses in 87.5%.

## Discussion

This mixed-method evaluation of the TSDP showed that nurse-led initiation and monitoring of ART patients was perceived by doctors, nurses and patients as feasible and acceptable in the Namibian context in comparison to care provided by doctors. Patients reported confidence in well-trained nurses’ capacity to provide them with appropriate HIV care and treatment. Patient perspectives on nurse capacity could influence their retention in care and treatment. Health workers favored the TSDP because they believed it improved nursing skills, clinic flow, and patient quality and access to care. Health care worker positive perspectives on task shifting implementation are crucial to successful implementation. Without buy-in and motivation to the proposed change in service delivery, increased capacity to provide care as a result of training may not result in increased provision of care. Health care worker and patient perspectives on task shifting advantages were very consistent with each other and similar to those reported elsewhere [32], [33].

The structured observations provided quantitative data which compared quality of care between doctors and nurses. There was no pre-determined level of acceptability by the MoHSS, however, the results of data collected in their own country showing high levels of agreement (>90%) on eight of the quality of care dimensions were considered encouraging. Detailed field notes kept by physician-evaluators were reviewed by senior Namibian clinicians for dimensions of care where doctors performed better than nurses in over 10% of cases. They concluded that additional clinical mentoring support could reasonably be expected to quickly improve nursing skills in those areas where doctors outperformed nurses.

Our methods enabled us to describe performance by doctors and nurses whose care was being observed and carefully annotated. Considering the increasing demand for care and treatment across Namibia, and the limited supply of doctors, a more realistic comparison might be between care and treatment by nurses or care and treatment provided by an overextended physician, often required to provide rushed care, many miles away from where the patients live. In some instances, the appropriate comparison might be between nurses providing care and treatment, to the patient receiving no care and treatment at all [34].

Our rapid assessment method presents several advantages for future studies in similar contexts, when time and resources for data collection are constrained. First, to our knowledge, only one other study has used direct observation to assess task shifting effectiveness. In Mozambique, non-physician clinicians (*técnicos de medicina)* were directly observed delivering HIV care (N = 127 patient encounters) after a two-week HIV/AIDS training and compared to the gold standard of expert clinical evaluators. [29] Agreement was well below 80%, prompting revisions in both the national in-service training strategy and the pre-service training curriculum for *técnicos de medicina.* What was not assessed in that study was whether the care provided by the *técnicos* was worse than routine care provided by doctors. As an improvement to that study’s methods, we compared nurses directly with doctors providing care at the same site or in the same catchment area, in addition to comparing both the nurse and the doctor to the physician- evaluator, adding value to our results and to the literature. In our study, agreement between nurses and doctors was higher than 80%, and our methodology enabled us to identify areas where additional support in clinical decision making for both nurses and doctors might be needed (e.g. assessing treatment failure, targeted physical exams).

Moreover, our direct observation method presents several advantages over implementation research studies on task shifting that used medical record abstraction or routine facility level data. [7], [18], [35]–[37] First, direct observation allows for determination of the type of care provider in settings such as ours where routine documentation to distinguish doctor from non-doctor led care was unavailable and it is impossible to use patient records to determine the cadre of care provider for comparison. Furthermore, direct observation as employed in our study can help distinguish correct diagnosis/care/treatment from correct documentation. For example, routine data can tell if a patient was staged, what the stage was, and whether ART was started as indicated. However, by looking at routine data one cannot tell whether the patient was staged correctly, i.e. whether the health care provider caught the clinical symptoms that would indicate the correct stage. Through direct observation by a trained physician-evaluator, an assessment can determine whether the healthcare professional both acted according to guidelines and recorded the information according to guidelines. In addition, notes from clinical evaluators describing disagreement provided helpful contextualization for assessing patient risk and provided useful information to target additional mentoring. While patient clinical outcomes make the strongest case for task shifting, both prospective and retrospective clinical outcome data required more time than the MoHSS had to make crucial decisions about how to reach its dispersed patient population. Other studies using clinical outcomes and directly comparing clinical outcomes for patients initiated and treated by doctors compared to those treated by nurses required between two and five years. [10], [19], [32], [38], [39] In instances where appropriate documentation, time frame, resources, or routine service delivery models do not allow for such data to be collected, direct observation of service delivery may serve as a feasible alternative.

### Moving from Demonstration to Scale-up

While nurses, doctors, and managers were enthusiastic about the benefits of task shifting, and there was encouraging evidence of comparable care, the TSDP also identified potential barriers. Anticipated challenges for national roll-out can be broadly categorized as: strengthening and aligning infrastructure to the new health service delivery model, providing ongoing mentoring and support of nurses, and revisiting the national nursing scope of practice.

First, several infrastructure barriers need to be addressed. At the time of the demonstration, files for patients visiting outreach clinics were stored in hospitals. If leaders roll out task shifting widely, these files and filing systems would have to be transferred to clinics where patients would seek care. The absence of pharmacies at the clinic level would also cause challenges for secure drug storage and management. In addition, in some hospitals and health centers, where both nurses and doctors practice on-site, space is a limitation; if there are not enough consultation rooms for both a nurse and doctor to work concurrently, the value of having a nurse trained to examine and assess patients is reduced. Site-specific preparation efforts that identify possible storage and space considerations could ameliorate some of these barriers. As more tasks shift to ART nurses, other staff may need to take over some of their current responsibilities. Acknowledging these systems level changes and the time and support they will take to implement will be important for the MoHSS’s planning purposes for task shifting scale-up.

Second, ongoing clinical mentoring was reported as an important component of this task shifting intervention, as has been reported elsewhere in the task shifting literature [9]–[12], [18], [40]–[42]. Due to the wide variation in distances, facilities, and staffing within regions, the Namibian Regional Health Management Teams will need to identify and test the best model of mentoring and supervision for their region. Where in person mentoring isn’t possible due to distances or over-extended physicians, a phone consultation model for scale up may be more feasible. [43] Senior nurse-to-junior nurse mentoring models have been implemented in a similar project in Ethiopia.[Unpublished data] These and other mentoring or supportive supervision approaches could be modified and tested for future use in the Namibian context.

Third, as identified elsewhere [9], [17], [32], national-level policy changes need to take place to maximize the impact of task shifting. The current nursing scope of practice does not allow for initiation of patients on ART; without a modified scope, nurses could face punitive and legal action for mistakes related to HIV care and treatment. As of this writing, conversations are in process between the MOHSS and the Namibian Nursing Council to revise the nursing scope of practice around ART delivery, a critical step for further expansion of this pilot and eventual nationwide scale-up.

Despite its small sample size, limited budget, and time constraints, our results compare favorably with large studies of nurse initiated treatment conducted region [18], [19], [37], [39], [40], [44], contributing to the task shifting and implementation science literature. Within the HIV/AIDS context in sub-Saharan Africa, the heterogeneity of affected populations, the context of expanding care and treatment, and the importance of sustainability make the operational research process of “learning by doing” advantageous. This hands-on approach may be the most flexible way to determine what methods or approaches would be best to increase ART access and develop robust responses to AIDS in various contexts across a continent [26]. This small-scale approach based on experiential learning had several benefits [45], [46] that ultimately increased the likelihood of acceptance of the new approach to HIV+ patient initiation and care: 1) the MoHSS was able to test the model with fewer risks allowing an opportunity to reverse course if findings were unfavorable, 2) it helped to identify benefits and constraints to scale-up in the Namibian context, 3) the process helped build group expertise if the decision was to move forward with expansion of the approach, and 4) the process promoted local adaptation of the intervention to increase the likely success and sustainability of the initiative.

### Limitations

This study has several limitations that may affect the results. The quality of observed care may be higher than usual care because health care workers may perform at a higher level under observation. However, this potential Hawthorne effect would be applicable to both doctors and nurses. The small number of observations may not be representative of the wide range of cases encountered in hospitals, health centers, and clinics in Namibia. In addition, the nurses, facilities, and doctors who participated in the TSDP were specifically identified by the MoHSS and their performance and enthusiasm for task-shifting may not be representative of other nurses and doctors in Namibia. Assessing how care provided by doctors and nurses nurse compared to national guidelines and to each other may not reflect clinical outcomes, such as survival, viral failure, and CD4 levels.

There may be some courtesy bias by the interviewees; however, the documentation of specific examples to illustrate broad opinions limits this likelihood. The identification of various constraints to task shifting also suggests minimal courtesy bias from health care workers and managers.

## Conclusion

The demand for HIV care and treatment in Namibia will continue to increase as various programmatic manifestations of “treatment as prevention” are developed and implemented over the next few years. This expansion will amplify the burden on an already overstretched health care system. The demonstration project enabled the MOHSS to adapt a “learn by doing” approach, i.e., to test the model on a small scale, providing an opportunity to allow for modification of the intervention as necessary before expansion. The findings from this small-scale Task Shifting Demonstration Project confirm that nurse task shifting for ART initiation in Namibia is an appropriate, indeed vital, policy in continuing the scale-up of life-saving HIV clinical services in Namibia. The implementation research process has given national HIV stakeholders in Namibia confidence in this new paradigm, while contributing to the growing regional evidence base. Once a nursing scope of practice has been revised, the Namibia MOHSS can build upon this approach through a phased roll-out. In this way, challenges can be resolved as they are identified and both quality of and access to care can be supported.
